# COVID-19: Reflections

**DOI:** 10.1017/dmp.2020.191

**Published:** 2020-12

**Authors:** James J. James

*Another such victory over the Romans, and we are undone*. – Pyrrhus

## CONTAINMENT

By the time air travel restrictions to the United States were put in place in January of this year, some 430 000 passengers had arrived at US airports on flights from China after the first cases of coronavirus disease (COVID-19) were identified.^1^ In addition to this, many more thousands of travelers continued to arrive from Europe (fast becoming the epicenter of the pandemic) through mid-March when case maps already showed the virus to be present in every US state and territory, and community transmission already established.^2^ As a public health intervention, containment was a non-starter; the virus had not only escaped, but also it was embedded across both the country and the globe.

## MITIGATION

These strategies involve a spectrum of possible interventions ranging from frequent handwashing and social distancing to school and business closures and mandatory stay-at-home orders. All of these come at a cost with closures and stay-at-home orders having the most severe socioeconomic impact versus the negligible costs of lesser measures. Given our commitment to science, we want to be able to demonstrate an evidentiary basis for our policies and not adopt costly interventions on opinion and anecdote. A summary of 2 systematic reviews on these strategies certainly supported social distancing measures but indicated no significant benefit from the more extreme ones.^3^ Nevertheless, as of mid-March, many countries around the world went into extreme lockdown mode, including a good number of US states. The stated intent was to “flatten the curve” in order to reduce pressure on medical systems; later, the focus shifted to saving individual lives. However, in the absence of a vaccine, this strategy does not prevent new cases, and it only defers them.

## RESULTS

On March 17, the United States reported some 300 000 cases of COVID-19 and 8000 deaths. Now, just 2 months later, we have over 1.5 million cases of COVID-19 and 100 000 associated deaths; and the epidemiological curve is only now starting to decline, in spite of the relatively short (5-day average) incubation period of the virus. While there is a general consensus that extreme lockdowns worked, this conclusion is based primarily on models of questionable validity and scant evidence. However, there is ample observational evidence that less extreme social distancing measures were non-inferior in mitigating COVID-19. Support for this conclusion can be found in studies^4,5^ for comparisons across nations and the United States. It is a logical assumption that extreme lockdowns should be the most effective mitigation strategy, but, in reality, this proves not to be the case. The most fundamental reason for this is that, once the virus is already prevalent in a community, you cannot completely prevent its spread. Even under the strictest quarantine, essential services must continue to be provided and essential items, such as food, must continue to be transported, thereby potentiating exposure and transmission. A more subtle flaw in our institution of extreme control measures, especially with reference to school closures, is that these strategies are based on evidence from influenza mitigation efforts, and we are dealing with a coronavirus that has markedly different epidemiological characteristics. Finally, and possibly most importantly, the shelter in place strategy might in fact increase risk of infection, in some cases. There is increased risk to other household members when we confine known and suspected cases at home, a risk that increases over time with active viral shedding. This could well account for the findings from a survey of 1269 New York City (NYC) hospitalizations, among whom 83% were retired or unemployed and had been at home for many weeks.^6^

## NUMBERS, STATISTICS, AND MODELS

One of the hallmarks of this pandemic has been the plethora of data collected and reported from around the world. This has enabled the generation of a myriad of descriptive calculations and a broad range of conclusions on the parameters of the disease and the interventions adopted to mitigate and control the pandemic. Unfortunately, (1) the great variability in transmission characteristics across and within countries; (2) significant demographic differences as to relative risk; (3) a lack of standardization and definition as to diagnostic criteria, testing and clinical triage protocols, and medical and public health capabilities; and (4) a serious lack of knowledge regarding agent characteristics and basic comparative studies to assess best practices are insufficient at best. This disarray in data sets has enabled the statistical support of any number of often conflicting conclusions. Further, and far more troublesome, these disparate data sets have served to produce the “estimates” for the predictive epidemiological models that have informed the extreme mitigation policies that are of questionable effectiveness but are very socioeconomically damaging. For reliable predictive modeling, 2 parameters must be well defined: the risk of acquiring an infection and the risk of transmitting the infection. Each of these is poorly understood with COVID-19, resulting in predictive outputs with variances too extreme to be useful, especially when the worst case becomes the banner headline for the media.^7^

## PANDEMICS AND RISK

*Courage is not the absence of fear but the triumph over it.* – Nelson Mandela

**FIGURE 1:**
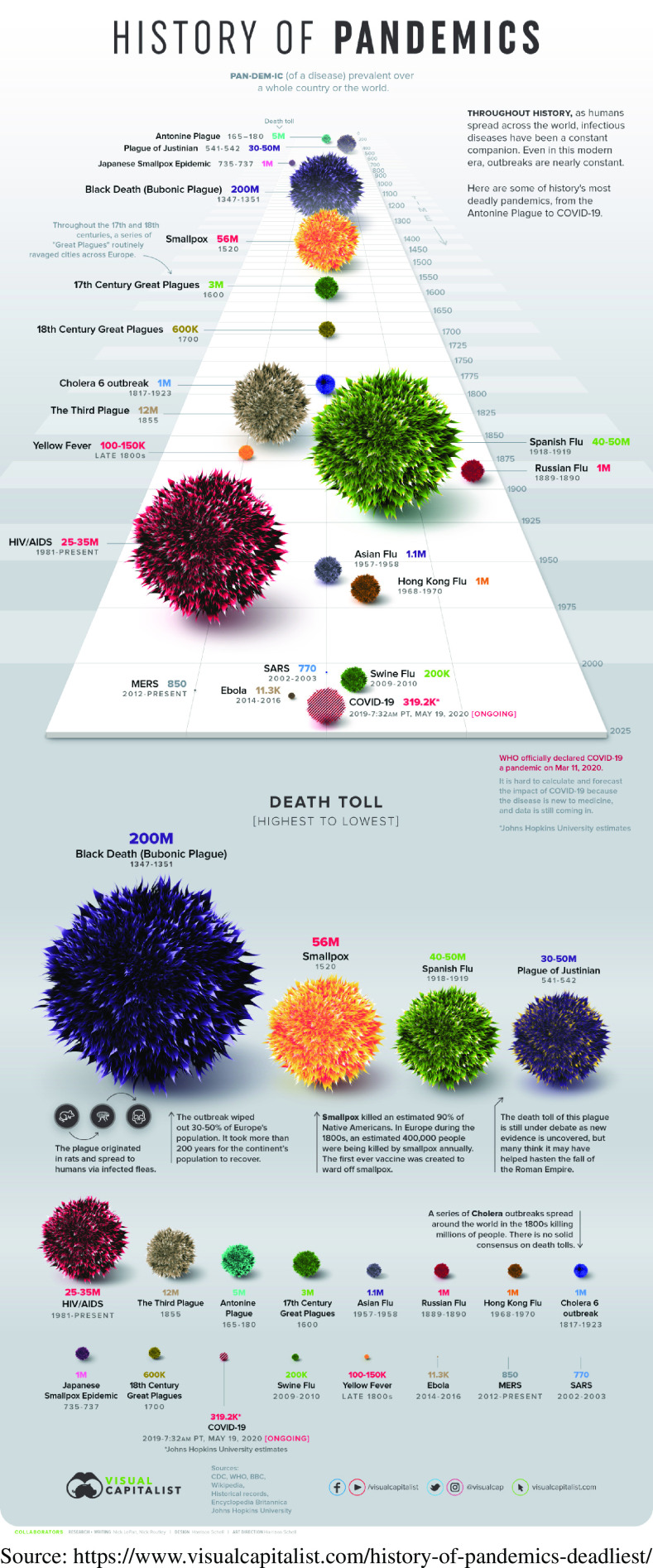
Historic Pandemic Mortality.

On March 11, 2020, the World Health Organization declared COVID-19 a pandemic, setting off a renewed wave of travel restrictions, quarantines, school and business closures, and elevating an already exaggerated level of fear around the globe. Unfortunately, pandemic is not only an emotionally charged word, but it is also used in an all-or-none sense. For other disasters, such as hurricanes and earthquakes, there are quantitative scales that help put things in perspective and allow more objective individual and community risk assessment. One thing that our experience with COVID-19 has made clear is the need to develop a pandemic scale to inform future declarations. Such a scale could assist in achieving levels of concern commensurate with individual risk for COVID-19. Looking at the following infographic^8^ (Figure 1), the first thing to note is that mortality has always served as the accepted comparative measure for assessing overall pandemic impact:

Using this measure, COVID-19 is but a spec on the chart; and, if the raw counts were expressed as population mortality rates, the differentials would be greatly magnified. Contrary to this precedent, we are using case numbers to define the current pandemic, even given the extreme variability in measuring case counts within and between countries. This practice maintains and intensifies population fear levels for a variety of conceivable reasons, none of which serve the public’s health. As a result of this, case counts coupled with worst-case modeling estimates create the headlines we are exposed to every day and maintain the heightened fear among the population. Interestingly, the 1957 and 1968 flu pandemics are represented in Figure 1, and each is more prominent than COVID-19, at least up to this point in time. At the height of the 1968 outbreak, *The New York Times* described the pandemic as “one of the worst in the nation’s history,” yet businesses and schools, for the most part, stayed open and there was little of the public hysteria that we see with COVID-19. One contributing factor was that, at the time, unlike today, most newspapers and publishers behaved responsibly and were reluctant to stoke public fears.^9^

Putting the risk of COVID-19 to date into proper perspective, even using case counts, the overall population attack in the United States today is 0.4%, and the mortality rate is 0.0003%. When further adjusted for age and underlying conditions, the risk of death for healthy Americans under the age of 65 approaches 1 in 20 000. None of this is meant to diminish the impacts of COVID-19 on thousands of Americans. It is a virulent and deadly disease that can be horrific for those afflicted and their loved ones, and it has had devastating impacts on health care systems and medical personnel. The purpose of presenting these numbers is to temper harmful levels of fear and to put COVID-19 into a more balanced perspective with regard to individual and community risks, so that we can better mitigate its medical consequences while preserving our socioeconomic infrastructure.

## VACCINE AND HERD IMMUNITY

*We hope to have a vaccine (for AIDS) ready … in two years.* – Margaret Heckler, HHS Secretary, 1984

Many of the proponents of continuing extreme lockdowns do so awaiting the fielding of a vaccine or an effective pharmacological intervention. This becomes problematic for a host of reasons: (1) From a global perspective, the socioeconomic damage will be unacceptable, (2) population unrest will increase, (3) as noted previously, there is no direct evidence to support added benefit when compared with less restrictive measures, and (4) most importantly, a safe and effective vaccine available in sufficient quantities is probably at least a year away under the best of circumstances.^10^

In the absence of a vaccine, we need to explore the concept of herd immunity. Herd immunity as a mitigation strategy has been dismissed up until now, because if utilized, it is thought of as a corollary to vaccination. The presumption is that achieving herd immunity in the absence of a vaccine would be extremely costly in terms of lives lost. This latter concern, however, might prove to be exaggerated. The dire predictions in terms of lives lost are based on 2 assumptions that may well be invalid: (1) We would need to achieve a level of 60%–70% of non-susceptible individuals in the population, and (2) over 90% of the US population remains susceptible to infection and must be protected from exposure.^11^ As to the first assumption, the herd immunity level is calculated from the estimated Ro, which is dependent on the number of susceptible individuals in a population. The estimated Ro for COVID-19 is between 1.4 and 3.9, which yields herd immunity at a level between 29% and 74%. The realistic target level is probably somewhere in the middle of that range but, more importantly, may well be achievable without a vaccine. It is also important to understand that, even if the actual level is not attained, any increase in the overall non-susceptible pool will help decrease the transmission rate and help flatten the curve.

## EXPOSURE

Issues surrounding exposure to and transmission of an infectious dose of COVID-19 are among the most critical to better our understanding of the epidemiology of this pandemic and probably the least understood parameters of this pathogen. Given our knowledge of the coronaviruses (ie, family of viruses), in general, there is little doubt that the primary mode of transmission is respiratory droplets. Other mechanisms most likely coexist, but their relative importance is difficult to measure. However, regardless of the transmission route, the critical questions revolve around the cumulative dose required to produce infection and how variable that dose is across different individuals. Evidence-based answers to these questions are not yet available, but a review of available data allows for plausible inferences to be made. Given the clustering of cases that is a hallmark of this disease, continued close contact, especially in closed spaces, leads to relatively high attack rates given the presence of an active shedder. This has been too frequently documented in funeral services, weddings, social gatherings, close contact work areas, medical facilities, and the whole spectrum of eldercare facilities. This would certainly imply that close contact over time in closed spaces is the highest risk setting for transmission. This should, again, bring into question the wisdom of confining actual and suspected cases with multiple family members in a closed space for prolonged periods of time. This concern is further amplified given that some 50% of PCR positives are subclinical or preclinical and is especially concerning in more confined living areas housing multi-generational families – conditions more often experienced by the more disadvantaged members of our society.^12^

## SUSCEPTIBILITY

Given exposure, the next issue is individual susceptibility. Are we all susceptible and are we equally so? These are parameters that are likewise far from being fully understood, but there are available data from which to draw inferences. An examination of the attack rates in the epicenters, such as NYC and Wuhan, raises a very legitimate question: why were the population attack rates so low? The easy answer is that exposure rates were limited, but this is more assumption than fact. Increasing evidence accumulated from wider testing demonstrates that even with exposure, attack rates are relatively low for what has been described as a highly infectious pathogen. The complete testing of the Diamond Princess and the Theodore Roosevelt yielded remarkably similar results. Combining the results, approximately 80% of the potentially exposed passengers and crew were PCR negative in an environment very conducive to transmission.^12^

For NYC, a follow-on serology survey showed an overall positivity rate of 20%, which strongly suggests that a fairly large number of residents had been exposed without becoming infected, or identified as PCR positive.^14^ Classically, infectious disease epidemiological models define 3 population categories: Infected, Recovered (immune), and Susceptible. This implicitly defines all those not infected or recovered as being at risk of becoming infected, if exposed. If this were the case with COVID-19, the overall population exposure versus the positive PCR and serology rates would be difficult to reconcile. This brings up the consideration that, for COVID-19, there is a fourth population category, which has been identified as Exposed Uninfected (EU). This relative immunity has been demonstrated in a variety of conditions to include COVID-19 and is attributed to genetic variants and their expression through our immune systems.^15^ The possibility of natural immunity in a significant percentage of the population further contributes to the potential of achieving herd immunity without the excessive loss of life previously predicted.

## WHAT TO EXPECT

The previous issues are vitally important because so many of the worst-case scenarios and dire predictions of second and third waves are based on the assumption that there remains a large pool of fully susceptible individuals who, upon exposure, will go on to become infected and that, in the absence of a vaccine, full lockdowns will have to be reinstituted. As to the size and timing of any future resurgences, given our current state of knowledge on COVID-19, no one can be certain regarding the number of infections that we will see. Wider PCR and serological testing will go a long way to providing us with the information we need to make more informed decisions and more effective policies; however, we must be cognizant of 2 considerations: (1) Given the large number of subclinical infected, increased PCR testing will itself produce an artificial increase in PCR-positive cases, and (2) as the prevalence of COVID-19 increases in the population, the predictive value of serological testing will improve. So, what can we expect and how should we proceed? We cannot be sure, but putting predictive models and worst-case scenarios aside, the following seems reasonable based on what we know and what we have experienced to date. COVID-19 is too deeply imbedded in the human ecosystem to go away anytime soon, if at all. More likely, it will join the host of other infectious diseases that we have learned not to accept, but to cope with. In the next few months, we need to maintain the sensible social distancing measures that have proven effective until we have an effective treatment or vaccine available. Until then, we have to grapple with the concept that the best way to protect our high-risk populations is through achieving a protective level of herd immunity, which involves accepting a level of increased test positives among those at minimal risk. Perhaps then we can return to the “old normal” with minimal damage to our overall well-being.
